# Inhibition of telomerase activity and induction of apoptosis by *Rhodospirillum rubrum* l‐asparaginase in cancer Jurkat cell line and normal human CD4+ T lymphocytes

**DOI:** 10.1002/cam4.1218

**Published:** 2017-10-05

**Authors:** Dmitry D. Zhdanov, Vadim S. Pokrovsky, Marina V. Pokrovskaya, Svetlana S. Alexandrova, Mikhail A. Eldarov, Dmitry V. Grishin, Marsel M. Basharov, Yulia A. Gladilina, Olga V. Podobed, Nikolai N. Sokolov

**Affiliations:** ^1^ Institute of Biomedical Chemistry Pogodinskaya st., 10/8 Moscow 119121 Russia; ^2^ N.N. Blokhin Cancer Research Center Kashirskoe Shosse 24 Moscow 115478 Russia; ^3^ Peoples’ Friendship University of Russia RUDN University Miklukho‐Maklaya st., 6 Moscow 117198 Russia; ^4^ Research Center of Biotechnology RAS Leninsky prospect, 33 Moscow 119071 Russia

**Keywords:** Antitumor activity, apoptosis, cell cycle inhibition, l‐asparaginase, replicative senescence, telomerase

## Abstract

*Rhodospirillum rubrum* L‐asparaginase mutant _E149R, V150P, F151T_ (RrA) down‐regulates telomerase activity due to its ability to inhibit the expression of telomerase catalytic subunit hTERT. The aim of this study was to define the effect of short‐term and long‐term RrA exposure on proliferation of cancer Jurkat cell line and normal human CD4^+^ T lymphocytes. RrA could inhibit telomerase activity in dose‐ and time‐dependent manner in both Jurkat and normal CD4^+^ T cells. Continuous RrA exposure of these cells resulted in shortening of telomeres followed by cell cycle inhibition, replicative senescence, and development of apoptosis. Complete death of Jurkat cells was observed at the day 25 of RrA exposure while normal CD4^+^ T cells died at the day 50 due to the initial longer length of telomeres. Removal of RrA from senescent cells led to a reactivation of hTERT expression, restoration telomerase activity, re‐elongation of telomeres after 48 h of cultivation, and survival of cells. These findings demonstrate that proliferation of cancer and normal telomerase‐positive cells can be limited by continuous telomerase inhibition with RrA. Longer telomeres of normal CD4^+^ T lymphocytes make such cells more sustainable to RrA exposure that could give them an advantage during anti‐telomerase therapy. These results should facilitate further investigations of RrA as a potent anti‐telomerase therapeutic protein.

## Introduction

Telomerase is a protein complex responsible for maintaining telomeric repeats TTAGGG at the end of human chromosomes. The main components of telomerase are hTR (human telomerase RNA), comprising RNA template for synthesis of telomeres, and hTERT (human telomerase reverse transcriptase) which possesses reverse transcription activity and synthesizes the telomeric repeats on the hTR template 1. Telomerase is active in normal reproductive cells, stem cells, activated lymphocytes, and most tumor types 2 and maintains the lifespan of malignant cells. It is known that the enzyme activity is mostly regulated by hTERT synthesis levels 3.

In normal somatic cells, telomerase is inactive, their telomeres are reduced by 40–200 b.p. every division cycle, and cells are only able to divide a limited number of times. Reduction of telomeres to a critical length causes cell transition into so‐called replicative senescence when the proliferation slows down or stops, but the cells remain alive and metabolically active 4. Change in the telomere structure at minimal critical length causes loss of main functions and triggers damage of DNA, which leads to apoptosis 5. Most cancer cells (about 90%) have telomerase activity 2. Active telomerase in cancer cells does not fully restore the telomere length typical for normal cells (telomeres in cancer cells are significantly shorter than in normal cells) but maintains their size 6, 7. High expression of telomerase is a hallmark of cancer. Inhibition of telomerase in cancer cells leads to telomere attrition, lifespan limitation the of these cells, senescence, and apoptosis 8, 9, 10. Telomerase inhibitors have been expected to become promising agents for cancer treatment 11, 12.

L‐Asparaginases catalyze the hydrolysis of L‐asparagine to L‐aspartate and ammonia 13. For more than several four decades, L‐asparaginases from *Escherichia coli* (EcA) and *Erwinia chrysanthemi* (EwA) have been used in the treatment of acute lymphoblastic leukemia, but their therapeutic usage is limited by adverse effects 14, 15, 16, 17. Recently, a *Rhodospirillum rubrum* L‐asparaginase (RrA), which has two times lower molecular weight, and therefore, is less immunogenic than EcA and EwA, was isolated 18, 19. It was shown that RrA and its RrA _E149R, V150P, F151T_ mutant but no other commercially available L‐asparaginases can suppress telomerase activity in human T‐cell lymphoma Jurkat cells 20. Inhibition of telomerase activity by RrA expected to be an additional mechanism of anticancer activity of RrA, which has dual (anti‐asparaginase and anti‐telomerase) effect in one protein.

However anti‐telomerase activity of RrA may affect normal activated lymphocytes since telomerase is active in these cells 21. In present work, we studied the effect of RrA on telomerase activity and determination of lifespan of acute T‐cell leukemia Jurkat cells and normal human CD4^+^ T‐lymphocytes.

## Materials and Methods

### Studied L‐asparaginase

For all studies, RrA _E149R, V150P, F151T_ mutant was used. The upstream, downstream, and enzymatic properties of the studied enzyme were described in 18, 19.

### Cell purification, cultivation, and treatment with RrA

The study was approved by Ethical Committee of the Institute of Biomedical Chemistry; written informed consents were obtained from all participants. The blood from healthy 18–25‐year donors (*n* = 4) was collected in Vacuette K3EDTA tubes (Greiner Bio‐One, Kremsmünster, Austria). Peripheral blood mononuclear cells (PBMC) were isolated using Lympholite‐H (Cedarlane, Burlington, Ontario, Canada) density gradient centrifugation. CD4^+^ T cells were purified from PBMC using CD4 +  Human Isolation Kit (Miltenyi Biotec, Bergisch Gladbach, Germany) according to the manufacturer's instructions. CD4^+^ T cells were seeded at 5 × 10^5^ cell/ml and cultured in 25 cm^2^ flasks in cell medium RPMI 1640 (Thermo Fisher Scientific Inc., Waltham, MA) supplemented with 10% FBS (Fetal Bovine Serum, Thermo Fisher Scientific Inc., Waltham, MA), penicillin (50 U/mL; Sigma‐Aldrich, St. Louis, MO), and streptomycin (50 mg/mL; Sigma‐Aldrich) with 5 *μ*g/mL anti‐CD28 (eBioscience Inc., San Diego, CA), 5 *μ*g/mL anti‐CD3 MAbs (MedBioSpectr, Moscow, Russia), and 100 U/mL rHu IL‐2 (R&D Systems, Minneapolis, MN). Cells were cultivated in 5% CO_2_/95% air in a humidified atmosphere at 37°C and re‐stimulated each 3 days with complete medium supplemented with IL‐2, anti‐CD3, and anti‐CD28 antibodies.

Acute T Cell Leukemia Jurkat cells (ATCC, Manassas, VA) were grown in RPMI‐1640 supplemented with 5% FBS, 50 U/mL penicillin and 50 mg/mL streptomycin and re‐stimulated each 3 days with complete medium.

All cytotoxic assays were performed in 96‐well flat‐bottom plates (Sigma‐Aldrich, St. Louis, MO) in a final volume of media 200 *μ*L/well. 1 × 10^5^ cells were incubated with different concentrations of RrA 19 for 72 h. Cell viability was determined by Lactate Dehydrogenase (LDH) activity in cell lysates using LDH Cytotoxicity Detection Kit (Takara, Mountain View, CA) 22 according to manufacturer's protocol. The standard index of IC50 was calculated for evaluation of RrA cytotoxicity in vitro. The maximal non‐toxic concentration of RrA was considered as a maximal concentration which induced insignificant cell death.

### RNA isolation and real‐time RT‐PCR

Total RNA from cells was extracted using RNeasy Mini kit from Qiagen (Valencia, CA) according to manufacturer protocol. Reverse transcription and real‐time RT‐PCR was performed as described by Basnakian and colleagues 23. 5 *μ*g of total RNA was reverse‐transcribed using RevertAid RT Kit (Invitrogen) in a 25‐*μ*L reaction mixture followed by real‐time RT‐PCR using CFX96 Touch^™^ Real‐Time PCR Detection System (Bio‐Rad, Hercules, CA). The reaction mix was prepared using Platinum SYBR Green qPCR Supermix‐UDG (Invitrogen) according to manufacturer recommendations using the following primers (5′‐3′).

hTERT sense: GTCCGAGGTGTCCCTGAGTA; hTERT antisense: CAGGGCCTCGTCTTCTACAG; 18S sense: GGATCCATTGGAGGGCAAGT; 18S antisense: ACGAGCTTTTTAACTGCAGCAA. Two‐temperature cycles with annealing/extension were used. The fluorescence was measured at the end of annealing step. Melting curve analysis was performed at the end of the reaction (after 45th cycle) between 60°C and 95°C to assess the quality of the final PCR products. The standard curves of reaction effectiveness were performed using four serially diluted samples (1:40, 1:80, 1:160 and 1:320) of each protein or 18S cDNAs. Levels of investigated genes expression were normalized relative to the expression of reference gene 18S. PCR products were separated in 1% agarose gel, stained with ethidium bromide, and photographed under UV light in a ChemiDoc^TM^ XRS imaging system (Bio‐Rad). Calculation of the relative RNA concentration was performed using CFX96 Touch software.

### Western blotting

Cells were lysed in 1 mL of TBE buffer (89 mmol/L Tris, 89 mmol/L H_3_BO_3_, 2 mmol/L EDTA, pH 8.0) by ultrasonic disruption (50 W, 2 min) using Sonic Dismembrator (Thermo Fisher Scientific Inc., Waltham, MA). Cell lysates were centrifuged for 10 min and 12,000*g* to remove cell debris. Protein in samples was measured using the Bradford protein assay (Pierce Biotechnology, Rockford, IL). Bovine serum albumin was used for serial dilutions for the calibration curve. The total protein extract from cells (50 *μ*g of total protein) was dissolved in 50 mmol/L Tris‐HCl, pH 6.8, 1% sodium dodecyl sulfate, 2 mmol/L EDTA, 1% 2‐mercaptoethanol, 7.5% glycerol, and denatured by heating at 100⁰C for 10 min. Proteins were separated in gradient PAAG 24 (100 V; 2 h), using NuPAGE^®^ Novex^®^ 4–12% Bis‐Tris Protein gels (Life Technologies, Carlsbad, CA). Proteins were transferred onto the nitrocellulose membrane in Novex transferring buffer (Invitrogen) at 40 V for 3 h. The membranes were stained with Ponceau S (Sigma‐Aldrich) 25. After soaking in the blocking solution Blotting‐Grade Blocker (Bio‐Rad) the membranes were incubated with monoclonal antibodies to glyceraldehyde‐3‐phosphate dehydrogenase (anti‐GAPDH) or anti‐hTERT (Abcam, Cambridge, MA) diluted 1:1000. Membranes were washed in Tris‐buffered saline, pH 7.6 with 0.1% Tween‐20 (Invitrogen) and incubated with secondary antibodies conjugated with horseradish peroxidase (Cell Signaling, Danvers, MA). Membranes were visualized using Super Signal chemiluminescent kit (Pierce Biotechnology) and documented in a ChemiDoc^TM^ XRS imaging system (Bio‐Rad). Relative amounts of proteins were determined by densitometry in GelAnalyzer 2010a (www.gelanalyzer.com).

### Telomerase activity assay

Telomerase activity was determined using (TRAP) Telomeric Repeat Amplification Protocol 2, 26. Cells were lysed in 10 mmol/L Tris‐HCl, pH 7.5, 1 mmol/L MgCl_2,_ 1 mmol/L EGTA, 0.1 mmol/L PMSF, 5 mmol/L 2‐Mercaptoethanol, 0.5% CHAPS и 10% glycerol (all from Sigma‐Aldrich) and centrifuged 30 min at 12,000*g*. Supernatants were stored at −80°C. Elongation of oligonucleotide substrate TS‐primer (Telomerase Substrate primer) (5′‐ AATCCGTCGAGCAGAGTT ‐3′) and followed amplification was conducted in 30 *μ*L of reaction mixture: 67 mmol/L Tris‐HCl, pH 8.8, 16.6 mmol/L (NH_4_)_2_SO_4_, 0.01% Tween‐20, 1.5 mmol/L MgCl_2_, 1 mmol/L EGTA (all from Sigma‐Aldrich), 0.25 mmol/L each of dNTPS (Syntol) and 2 *μ*L of cell lysate (equivalent of 2000 cells). Elongation was performed for 30 min at 37°C and 10 min at 96°C for telomerase inactivation. 0.1 *μ*L of CX‐primer (Copy Extended primer) (5′‐ CCCTTACCCTTACCCTTACCCTAA ‐3′) and 2.5 Units of Taq‐polymerase was added to elongation mixture followed by PCR reaction: 94°C—5 min; 30 cycles: 94°C—30 sec, 50°C—30 sec, 72°C—40 sec; 72°C—5 min. Visualization of PCR products was performed in 12% non‐denaturation PAAG electrophoresis and TBE buffer. 10 *μ*L of samples were added to each well of gel comb. Gels were stained with SYBR Green I (Invitrogen) and photographed under UV light in a ChemiDoc^™^ XRS imaging system and analyzed by GelAnalyzer 2010a.

### Telomere length detection

The method described by R.M. Cawthon et al. 27, 28 was used to determine telomere length. Genomic DNA was isolated using PureLink Genomic DNA Mini Kit (Thermo Scientific Inc.) and real‐time PCR was performed 28. Telomere length was measured triplicate in each sample. For further calculations mean telomere length was considered actual if the intra‐experimental coefficient of variation was not higher than 2%. DNA from non‐transfected cells was used for reference control.

### Detection of apoptosis and cell cycle

In order to measure apoptosis, transfected CD4^+^ T cells were treated with Trypsin and EDTA (Gibco), re‐suspended in PBS (Gibco) and incubated with Annexin V‐FITC and propidium iodide (PI) from FITC Annexin V/Dead Cell Apoptosis kit (Invitrogen) according to manufacturer protocol. Counting 5x10^4^ cells at each point was performed by flow cytometry.

To measure cell cycle cells were fixed in 70% ethanol and treated with FxCycle PI/RNase Staining Solution (Thermo Scientific Inc.) according to manufacturer protocol. Cell cycle was measured by flow cytometry upon detection of propidium iodide signal.

### Detection of *β*‐Gal activity

The activity of *β*‐Gal in cells was determined using Beta‐Galactosidase Detection Kit (Abcam) according to manufacturer protocol in 96‐well black plastic plate (Corning). Detection of fluorescence was assessed by plate photometer MultiscanGo (Thermo Scientific Inc.) in the spectra excitation and emission 490 nmol/L and 525 nmol/L respectively. Calculation of activity was assessed for 1 × 10^3^ cells based on the calibration curve obtained with commercial b‐Gal (Abcam) in the range 0.01–100 U/mL.

### Statistics

Statistical analysis involving Student's *t*‐test was implemented with the Statistica 6.0 software (StatSoft, Tulsa, OK). The differences described by *P* ≤ 0.05 were considered significant. The results are presented as mean ± standard error of mean (SEM).

## Results

### RrA cytotoxicity for cancer Jurkat and normal CD4^+^ T cells

**Figure 1:**
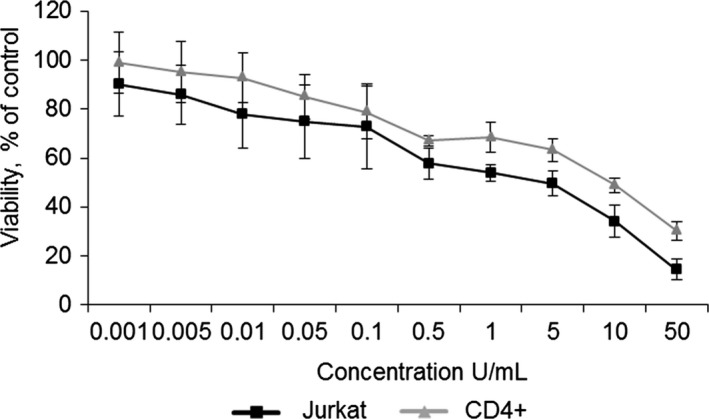
Cytotoxic activity of RrA for Jurkat and CD4^+^ cells. Jurkat and CD4^+^ T cells were co‐incubated for 72 h with various concentrations of RrA. Cell viability was determined using LDH assay. Data are presented as mean viability ± SEM. *N* = 4. *P* ≤ 0.05 was considered significant by Student's *t*‐test.

It is known that l‐asparaginase activity induces direct cytotoxic effect on cells 29, 30, while the effect of telomerase inhibition may develop for a longer period of time 31, 32. Cytotoxic activity of RrA was tested in 72 h of incubation with Jurkat cells or normal CD4^+^ T lymphocytes. RrA showed a dose‐dependent cytotoxic effect for both Jurkat and CD4^+^ cells (Fig. 1). Jurkat cells were more sensitive to high concentrations of RrA (1–50 U/mL) in comparison with normal CD4^+^. There was no significant statistical difference for cytotoxicity between Jurkat and CD4^+^ cells at low concentrations of RrA (0.001–0.5 U/mL). IC50 = 20.7 ± 1.0 U/mL for Jurkat cells and 29.5 ± 3.4 U/mL for CD4^+^ T cells. The value of RrA maximal non‐toxic concentration was 0.1 U/mL for both Jurkat and CD4^+^ T cells. We assumed that treatment of cells with RrA in such concentration would prevent direct toxicity of RrA due to asparagine deprivation.

### RrA suppresses telomerase activity at dose‐dependent and time‐dependent manner

**Figure 2:**
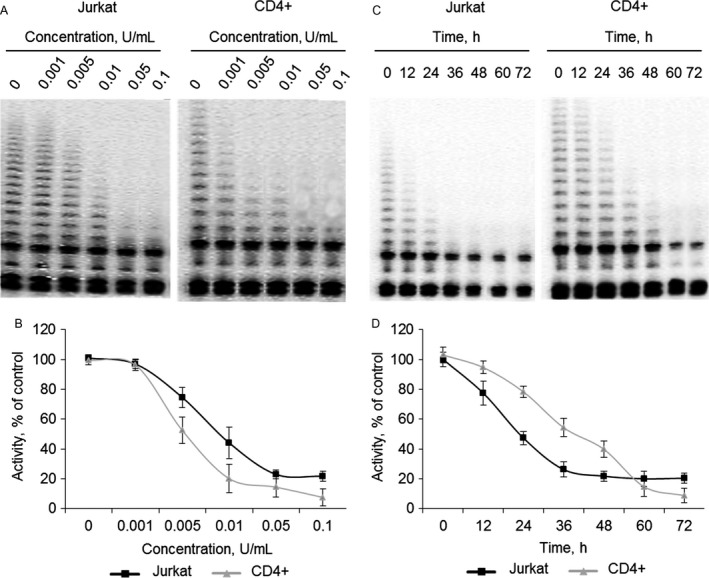
Dose‐dependent and time‐dependent ability of RrA to suppress telomerase activity. Jurkat and CD4^+^ T cells were incubated for 48 h with different concentrations of RrA or with 0.1 U/mL of RrA followed by detection of telomerase activity. (A,C) Telomerase activity determined by TRAP assay. (B, D) Results of TRAP quantification by densitometry. Data are presented as mean activity ± SEM. *N* = 4. *P *≤* *0.05 was considered significant by Student's *t*‐test.

We tested the ability of different concentrations of RrA to suppress telomerase in Jurkat and CD4^+^ cells at 48 h of incubation. RrA could suppress the activity of telomerase in a dose‐dependent manner in concentrations up to 0.05 U/mL for Jurkat cells and up to 0.01 U/mL for CD4^+^ cells (Fig. 2A and B). Incubation with higher concentrations did not lead to the additional reduction in telomerase activity. The residual levels were 22.9 ± 3.0% in Jurkat cells and 7.5 ± 5.7% in CD4^+^ cells.

We investigated the time‐dependent suppression of telomerase at maximal non‐toxic concentration 0,1 U/ml. RrA could gradually suppress telomerase activity during first 36 h of incubation up to 21.7 ± 6.2% in Jurkat cells and during first 60 h up to 14.7 ± 6.7% in CD4^+^ T cells (Fig. 2C and D).

### RrA does not suppress telomerase directly but down‐regulates hTERT expression

**Figure 3:**
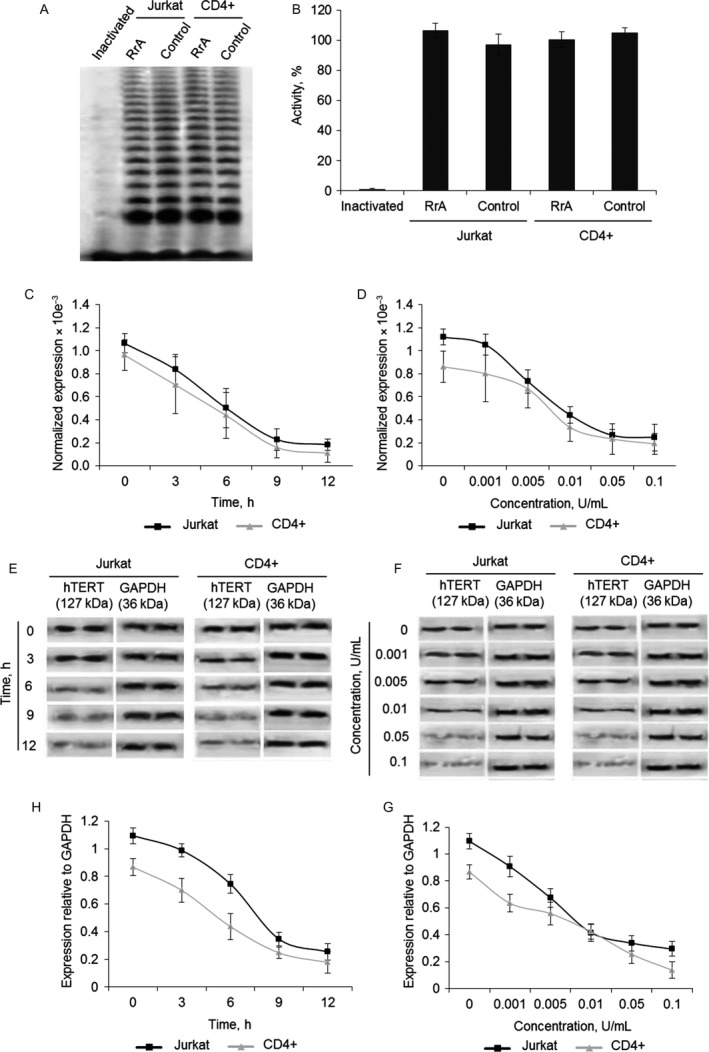
Ability of RrA to down‐regulate the expression of *hTERT* rather than to directly affect the activity of telomerase complex. RrA was added into TRAP assay to the final concentration 0.1 U/mL followed by detection of telomerase activity. (A) Telomerase activity determined by TRAP assay. (B) Results of TRAP quantification by densitometry. In order to investigate time‐dependent activity cells were incubated with 0.1 U/mL of RrA followed by detection of hTERT gene expression and protein quantification. To investigate dose‐dependent activity Jurkat and CD4^+^ T cells were incubated for 9 h with different concentrations of RrA. (C) Time‐dependent and (D) dose‐dependent expression of *hTERT* measured real‐time RT‐PCR in Jurkat and CD4^+^ cells. Levels of *hTERT* expression were normalized relative to the expression of reference gene 18S. (E) Time‐dependent and (F) dose‐dependent changes of hTERT protein amounts measured by western blotting. (G,H) Results of hTERT quantification relative to GAPDH. Data are presented as mean ± SEM. *N* = 4. *P *≤* *0.05 was considered significant by Student's *t*‐test.

One of the possible mechanisms for suppression of telomerase activity is the inhibition of telomerase complex itself. In order to determine the ability of RrA to inhibit telomerase directly, RrA was added to lysates of Jurkat and CD4^+^ T cells, and TRAP assay was performed. We found that RrA could not suppress telomerase activity in cell lysates (Fig. 3A and B). These results indicate the mechanism of telomerase suppression is different from direct inhibition of the telomerase complex.

Telomerase activity is strongly regulated by expression of its catalytic subunit hTERT 3. We investigated the expression of *hTERT* in Jurkat and CD4^+^ cells incubated with RrA at different time points using real‐time RT‐PCR. RrA was found to down‐regulate *hTERT* expression on time‐dependent manner at first 9 h of incubation in both Jurkat and CD4^+^ cells (Fig. 3C). Normalized *hTERT* expression at 9‐h time‐point decreased to 0.18 ± 0.05 × 10^−3^ in Jurkat cells and 0.17 ± 0.08 × 10^−3^ in CD4^+^ cells. Increasing the time of incubation up to 12 h did not significantly affect this level.

Perhaps, 9 h is a sufficient period for the down‐regulation of hTERT expression to the minimal level and is necessary for RrA to penetrate through cell and nuclei membranes and activation of suppressors of *hTERT* gene expression or binding regulate elements in promotor region of *hTERT* gene.

We investigated the expression of *hTERT* in cells incubated during 9 h with different concentrations of RrA. Dose‐dependent down‐regulation of *hTERT* expression was found in both Jurkat and CD4^+^ cells (Fig. 3D). *hTERT* expression dramatically decreased to minimal level 0.26 ± 0.05 in Jurkat cells and 0.23 ± 0.13 in CD4^+^ cells at concentrations 0.05 U/mL. Higher concentration of RrA up to 0.1 U/mL did not significantly affect this level.

Western blotting results showed significant reduction of hTERT protein in Jurkat and CD4^+^ cells incubated with RrA for different time (Fig. 3E and G) or with different concentrations (Fig. 3F and H).

### RrA induces the death of cancer Jurkat and normal CD4^+^ T cells

**Figure 4:**
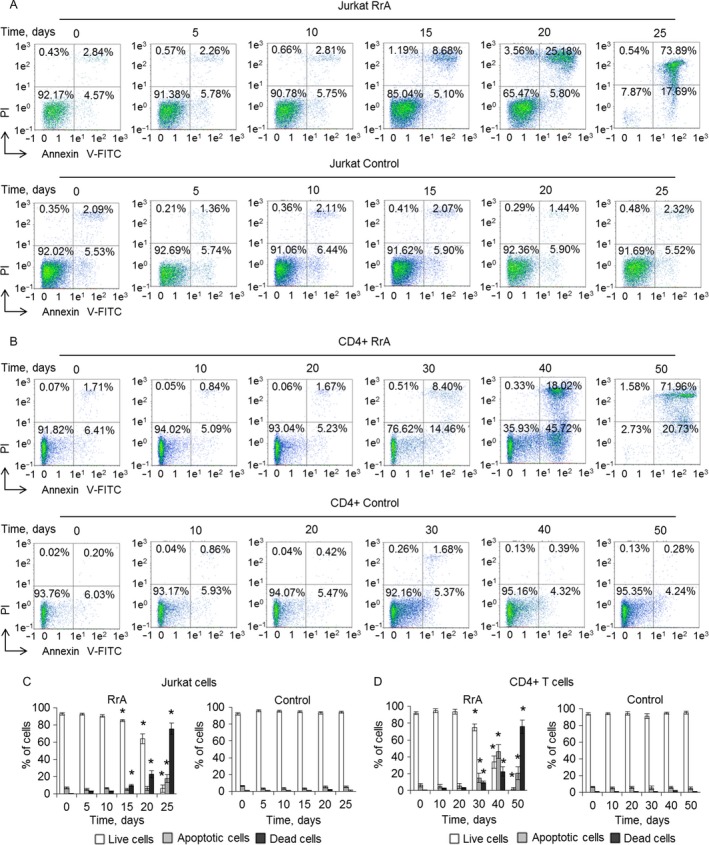
Induction of Jurkat cells’ and normal CD4^+^ T cells’ killing during cultivation with RrA. Flow cytometry results of (A) Jurkat cells and (B) CD4^+^ T cells incubated with RrA and labeled with Annexin V‐FITC and PI. Ratio of live cells (low left quadrants), apoptotic cells (low right quadrants), and dead cells (two upper quadrants) are presented. Histograms of live, apoptotic, and dead (C) Jurkat cells and (D) CD4^+^ cells. *N* = 4. *P ≤ 0.05 versus control cells.

Proliferation capacity of cells with inactive telomerase is of great interest since RrA induced down‐regulation of hTERT expression and caused the decrease of telomerase activity. To determine the proliferation capacity Jurkat and CD4^+^ T cells were co‐cultivated with 0.1 U/mL of RrA. The ratio of living, apoptotic, and dead cells was measured daily. The massive death of Jurkat cells was determined at the days 15–25 of cultivation (Fig. 4A and C). At day 25 almost all Jurkat cells were dead. The massive death of CD4^+^ cells was determined at the days 40–50 of cultivation (Fig. 4B and D). At day 30 dramatic increase of apoptotic CD4^+^ cell up to 13.3 ± 3.4% was observed. At day 50 almost all the cells were dead. Control Jurkat and CD4^+^ T cells stayed alive during all period of cultivation. The ratio of apoptotic and dead cells did not exceed control group.

### RrA converts cells to replicative senescent and inhibits cell cycle

**Figure 5:**
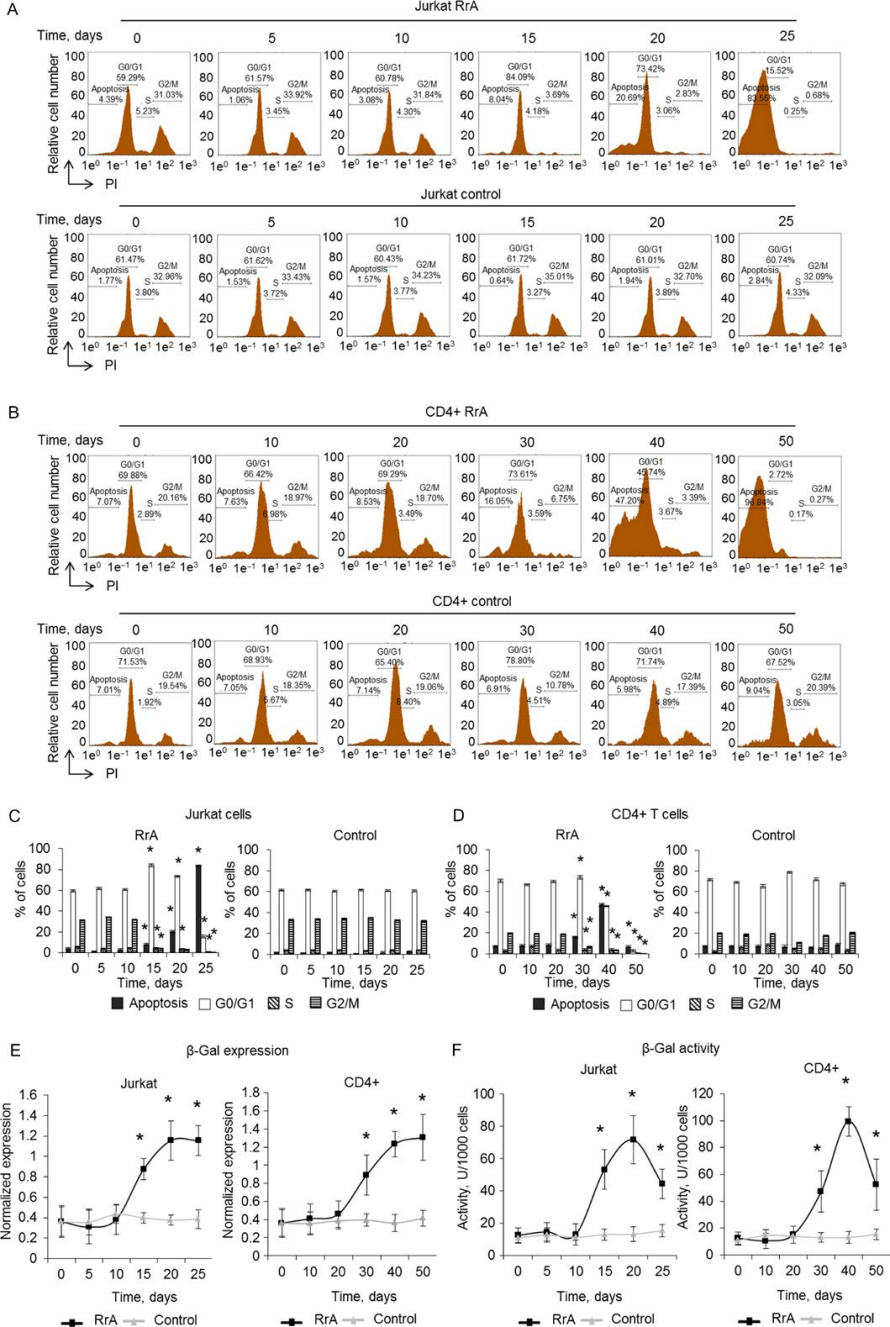
Inhibition of cell cycle and conversion of cells into the replicative senescent state. Results of cell cycle measured by flow cytometry and labeling of cell DNA with PI. Proportion of (A) Jurkat and (B) CD4^+^ cells in apoptotic state as well as in G0/G1, S and G2/M phases of cell cycle are presented. (C, D) Histograms of proportions of cells in different phases of cell cycle. (E) *β*‐Gal expression levels in Jurkat and CD4^+^ T cells cultivated with RrA. Levels of *β*‐Gal gene expression were normalized relative to the expression of reference gene 18S. (F) Enzymatic activity of *β*‐Gal in Jurkat and CD4^+^ T cells. *β*‐Gal activity is presented as U for 1000 cells which was determined by the calibration curve. *P ≤ 0.05 versus control cells.

Telomerase inhibition in proliferating cells is known to convert cells into replicative senesce, which associates with cell cycle arrest in G0/G1 phase 33, 34. We investigated cell cycle of proliferating Jurkat and CD4^+^ cells using flow cytometry and labeling cell DNA with propidium iodide. Cultivation with RrA led to significant reduction of Jurkat cells in S and G2/M phases and increase in G0/G1 phase on the day 15 (Fig. 5A and C). However, these cells stayed alive (Fig. 4A and C). At the days 15–25, consequent reduction of the number of cells in G2/M phase and an increase of apoptotic cells was determined. At day 25, almost all cells were in apoptosis. S and G2/M of cell cycle arrest were also observed in CD4^+^ T cells at days 30–50 of cultivation (Fig. 5B and D). At the 50th day, almost all cells developed apoptosis. Control Jurkat and CD4^+^ T cells did not demonstrate the changes in cell cycle during cultivation with RrA.

Induced expression and activity of *β*‐Gal is a marker of replicative senescent cells. We investigated expression and activity of this enzyme during cultivation. In Jurkat cells, RrA induced a significant increase of *β*‐Gal expression at the days 15–25 (Fig. 5E). Enzymatic activity of *β*‐Gal was elevated at day 15 of cultivation and remained high until day 20 (Fig. 5F). At day 25 the decrease of enzymatic activity was observed, which can be explained by massive cell death. RrA also up‐regulated the expression and activity of *β*‐Gal at days 30–50 of cultivation. A slight decrease of *β*‐Gal at day 50 was also observed due to cell death. In control, Jurkat and CD4^+^ T cells expression and activity of *β*‐Gal remained unchanged.

Activation of *β*‐Gal during cultivation with RrA is compliant with inhibition of cell cycle in S and G2/M phase, indicating replicative senescence of the cells. The further cultivation resulted, indeed, in a death of these cells.

### Replicative senescence associates with decreased expression of hTERT and the length of telomeres

**Figure 6:**
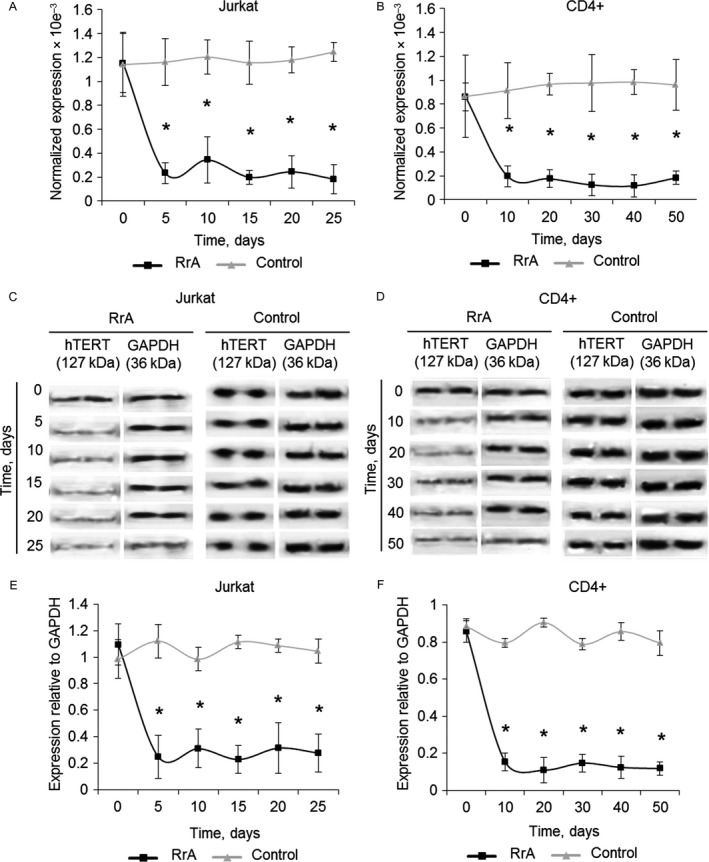
Decreased levels of *hTERT* expression in Jurkat and CD4^+^ T cells cultivated with RrA. Levels of *hTERT* expression in (A) Jurkat and (B) CD4^+^ T cells measured by real‐time RT‐PCR. Levels of *hTERT* expression were normalized relative to the expression of reference gene 18S. Western blotting of hTERT in (C) Jurkat cells and (D) CD4^+^ cells cultivated with RrA. (E, F) Results of hTERT quantification relative to GAPDH. *N* = 4. *P ≤ 0.05 versus control cells.

We investigated the changes in *hTERT* expression during cultivation of Jurkat and CD4^+^ T cells with RrA. Using real‐time RT‐PCR we determined the level of *hTERT* expression remained suppressed in Jurkat (0.24 ± 0.12 × 10^−3^) (Fig. 6A) as well as in CD4^+^ cells (0.12 ± 0.09 × 10^−3^) (Fig. 6B) during all time of cultivation. Control Jurkat and CD4^+^ T cells had a stable expression of *hTERT*. Significant changes of hTERT protein in Jurkat and CD4^+^ cells cultivated with RrA were confirmed by western blotting (Fig. 6C–F).

**Figure 7:**
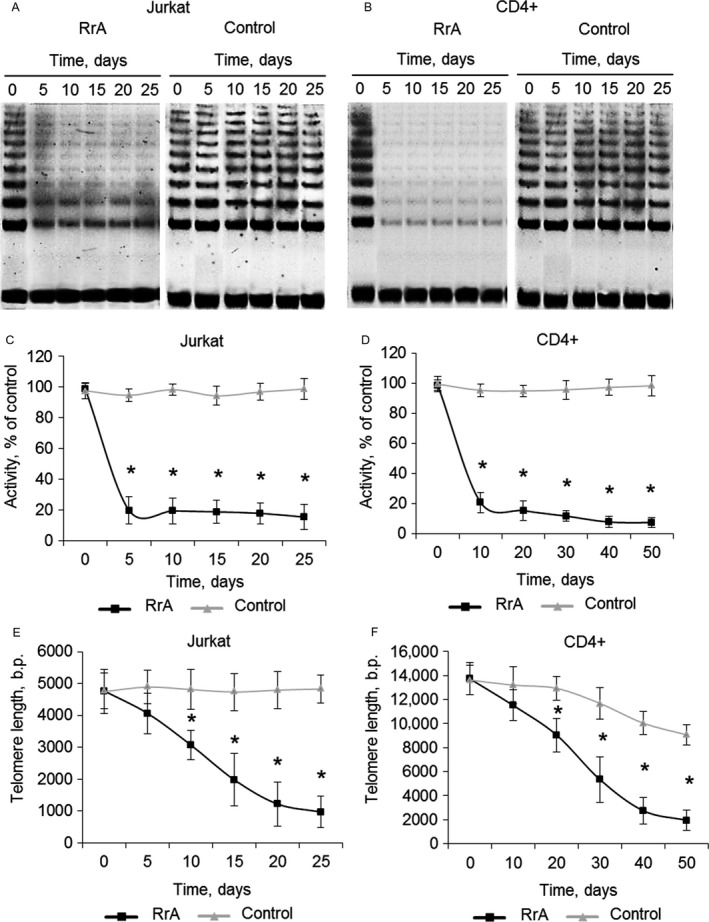
Decreased telomerase activity and the length of telomeres in Jurkat and CD4^+^ cells during cultivation with RrA. TRAP gel electrophoresis for (A) Jurkat and (B) CD4^+^ cells. (C,D) Results of telomerase activity measured by TRAP. Telomere length in (C) Jurkat and (D) CD4^+^ T cells measured by real‐time PCR. *N* = 4. *P ≤ 0.05 versus control cells.

Telomere shortening to critical length is known to be the reason for conversion of cells to replicative senescence. We investigated the changes in telomerase activity and telomere length in Jurkat and CD4^+^ cells during cultivation. Using TRAP assay we determined that both Jurkat and CD4^+^ cells had decreased telomerase activity during all period of cultivation (Fig. 7A–D). Control cells had a stable activity of telomerase.

The decrease of telomerase activity in proliferating cells should induce shortening of telomeres. Using real‐time PCR we showed that length of telomeres in Jurkat cells has been gradually decreasing from 4770 ± 567 b.p. to 1224 ± 687 b.p. during 20 days of cultivation (Fig. 7E). Control Jurkat cells had a relatively stable length of telomeres. The length of telomeres also decreased in proliferating CD4^+^ cells from 13731 ± 1342 b.p. to 1956 ± 835 b.p. during 40 days of cultivation (Fig. 7F). Longer cultivation resulted in further reduction of telomere length, but the rate of reduction was significantly slower both in Jurkat and CD4^+^ cells. In control CD4^+^ T cells reduction of telomere length was also observed, but it was not as rapid as in RrA‐treated cells. In control CD4^+^ T cells reduction of telomere length was also observed, but it was not as dramatic as in RrA‐treated cells. At day 50 of cultivation, the length of telomeres in control CD4^+^ cells decreased to 9672 ± 860 b.p.

### Cancer Jurkat and normal CD4^+^ T cells recover telomerase activity and survive after RrA removal

**Figure 8:**
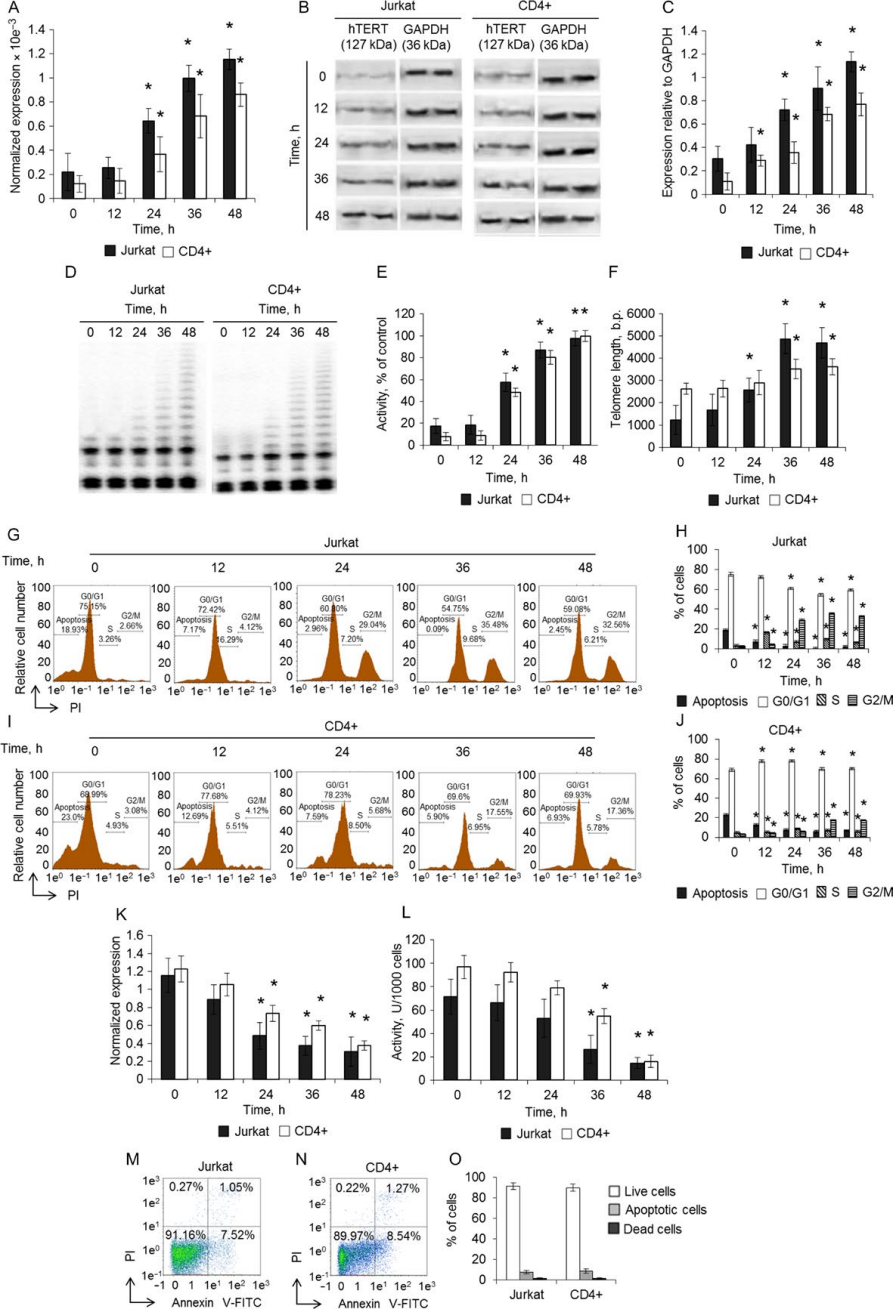
Restoration of telomerase activity and cell cycle after removal of RrA from media. (A) Expression of *hTERT* in Jurkat and CD4^+^ T cells after removal of RrA from cell growth media. Levels of *hTERT* expression were normalized relative to the expression of reference gene 18S. (B) Western blotting of *hTERT* in Jurkat and CD4^+^ T cells. (C) Results of hTERT quantification relative to GAPDH. (D) Detection of telomerase activity in Jurkat and CD4^+^ T cells by TRAP assay. (E) Results of TRAP quantification by densitometry. (F) Telomere length in Jurkat and CD4^+^ T cells measured by real‐time PCR. Results of cell cycle measuring using flow cytometry and labeling of cell DNA with propidium iodide. Proportion of (G) Jurkat and (H) CD4^+^ cells in apoptotic state as well as in G0/G1, S and G2/M phases of cell cycle are presented. (I, J) Histograms of proportions of cells in different phases of cell cycle. (K) *β*‐Gal expression and levels in Jurkat and CD4^+^ T cells. Levels of *β*‐Gal gene expression were normalized relative to the expression of reference gene 18S. (F) Enzymatic activity of *β*‐Gal in Jurkat and CD4^+^ T cells. *β*‐Gal activity is presented as U for 1000 cells which was determined by calibration curve. Flow cytometry results of (M) Jurkat cells at 25‐day time‐point (from Fig. 4A) of incubation in RrA‐removed media and (N) CD4^+^ T cells at 50‐day time‐point (from Fig. 4B). Cells were labeled with Annexin V‐FITC and propidium iodide. Proportion of live cells (low left quadrants), apoptotic cells (low right quadrants), and dead cells (two upper quadrants) are presented. (O) Histograms of live, apoptotic, and dead Jurkat and CD4^+^ T cells (C). Time‐point 0 h corresponds to the start of cell growth in RrA‐free growth media (the day 15 for Jurkat cells and the day 30 for CD4^+^ T cells). Data are presented as mean ± SEM. *N* = 4. *P *≤* *0.05 was considered significant by Student's *t*‐test. *N* = 4. *P ≤ 0.05 versus cells cultivated with RrA.

To exclude the possibility that the shortening of telomeres observed in RrA‐treated cells may have occurred as a consequence of insufficient RrA exposure, we examined the recovery of hTERT expression, telomerase activity, telomere length, cell cycle and cell death after removal of RrA from growth media. Jurkat cells at day 15 (from Fig. 4A) and CD4^+^ T cells at day 30 (from Fig. 4A) were switched to RrA‐free media. Cultivation of cells in the absence of RrA resulted in a gradual increase of *hTERT* expression during 48 h (Fig. 8A–C) as well as reactivation of telomerase (Fig. 8D and E). Significant re‐elongation of telomeres was observed in Jurkat cells after 24 h of growth in the absence or RrA (Fig. 8F), at 36 h of growth the length of telomeres restored completely. Re‐elongation of telomeres in CD4^+^ cells was not significant until after 36 h of growth, and the full length of telomeres in these cells has not been reached even at 48 h of growth in RrA‐free media. Along with re‐elongation of telomeres, we observed recovery of the cell cycle to the rate of non‐treated cells (Fig. 8G–J) and dramatic reduction of *β*‐Gal expression (Fig. 8K) and *β*‐Gal activity (Fig. 8L).

We measured the ratio of living, apoptotic, and dead cells by flow cytometry after removal of RrA from media. Jurkat cells were examined at day 25 (from Fig. 4A) and CD4^+^ T cells at day 50 (from Fig. 4B). Both Jurkat and CD4^+^ cells restored their lifespans (Fig. 4M–O), the number of living, apoptotic, and dead cells did not differ from control cells cultivated in RrA‐free conditions.

## Discussion

Telomerase is highly expressed in more than 85% of human tumors 35. Therefore, the development of agents, which are active against telomerase is a promising approach to develop novel cancer therapy.

In this report, we demonstrated that the immortal phenotype of blood cancer cells Jurkat can be reversed by continuous exposure to RrA. A potential side effect that could limit the clinical value of RrA due to its possible activity against normal telomerase‐positive cells such as proliferating lymphocytes. However, most of the cancer cells including Jurkat cells are known to have much shorter telomeres than normal cells 32, 36. These short telomeres were expected to make Jurkat cells and other malignant cells selectively sensitive to the effects of prolong telomerase inhibition.

Since hTERT protein is the catalytic rate‐limiting subunit of telomerase activity, several agents have been used to target hTERT, such as oligonucleotides, ribozymes, or small interfering RNA, which has been effective in knocking down expression 37. Our data showed that RrA could suppress hTERT expression and telomerase activity in both cancer and normal cells at time‐ and dose‐dependent manner (Figs. 2, 3). Unfortunately, we never observed 100% inhibition of hTERT expression and telomerase activity.

Telomere length plays important roles in maintaining genome stability and regulating cell replication and death 38. As expected for a telomerase inhibitor, RrA affected proliferation and survival only after a required sufficient telomere shortening (Fig. 7). We investigated the levels of telomerase activity in RrA‐exposed cells, and an unexpected finding was the small difference in hTERT expression and telomerase activity between cancer Jurkat cells and normal activated CD4^+^ T lymphocytes (Figs. 2, 3).

Cultivation of both Jurkat and CD4^+^ T cells led to the shortening of their telomeres to critical length and cell transition to replicative senescence, which is confirmed by elevated expression and activity of *β*‐Gal and by G0/G1 cell cycle arrest (Fig. 5). Telomere shortening upon cultivation was accompanied with a reduced expression of hTERT (Fig. 6) and decrease in telomerase activity (Fig. 7). Most probably, telomere length in the RrA‐exposed cells was reduced to the critical value, and cells entered replicative senescence. These cells remained alive, but they lost their ability to divide, which was confirmed by G0/G1 cell cycle arrest and reduction in a number of cells in S and G2/M phases (Fig. 5). Massive cell death (Fig. 4) was probably caused by the development of apoptosis in the senescent cells.

Telomere shortening during cell division was also observed in control untreated CD4^+^ T cells. These data are consistent with the limited replicative potential of T lymphocytes 39. Very low telomerase activity is detected in peripheral blood T lymphocytes 40. In vitro telomerase activity significantly increases upon stimulation of cell proliferation with IL‐2 and antibodies against CD3 and CD28 41. However, its activity is insufficient for unlimited proliferation of T cells 42.

These results are consistent with RrA limiting lifespan by the gradual shortening of telomeres. This explanation is strengthened by the observation that inhibition of hTERT expression with RrA is reversible. Removal of RrA from cultivation media restored most of these effects, including induction of hTERT expression, reactivation of telomerase, re‐elongation of telomeres, resolution of the cell cycle, and escape from the crisis within 48 h.

Taken together, our results showed that telomerase inhibition in cancer Jurkat cells and normal CD4^+^ T lymphocytes due to the long‐term exposure to RrA induced telomere shortening and eventual growth arrest and apoptosis in vitro. However, relatively short telomeres in Jurkat cells make these cells more sensitive to prolonged telomerase inhibition. Thus, telomerase inhibition using RrA may be considered as a promising approach for cancer treatment.

## Conflict of Interest

The authors declare no competing interests.
